# Alterations of gut microbiota in infants with biliary atresia identified by 16S rRNA-sequencing

**DOI:** 10.1186/s12887-024-04582-9

**Published:** 2024-02-14

**Authors:** Fei Liu, Ru Wei, Xiaogang Xu, Menglong Lan, Boyuan Tao, Zijian Liang, Jixiao Zeng

**Affiliations:** 1 Department of Pediatric Surgery, Guangdong Provincial Clinical Research Center for Child Health, Guangzhou Women and Children’s Medical Center, Guangzhou Medical University, 9 Jinsui Road, Guangzhou, Guangdong 510623 China; 2 Department of Children Health Care, Guangzhou Institute of Pediatrics, Guangzhou Women and Children’s Medical Center, Guangzhou Medical University, 9 Jinsui Road, Guangzhou, Guangdong 510623 China

**Keywords:** Biliary atresia, Gut microbiota, 16S rRNA-sequencing, Biomarker, Infant

## Abstract

**Background:**

Biliary atresia (BA) is a severe neonatal disease with progressive intra- and extra-hepatic bile ducts inflammation and hepatic fibrosis. Characterization of gut microbiome profiles in infants with biliary atresia can provide valuable information and potential disease biomarkers. Our study aims to explore the relationship between gut microbiota and biliary atresia.

**Methods:**

16 S ribosomal RNA (rRNA) gene sequencing was carried out to identify the differences in composition and diversity of gut microbiota between infants with BA and healthy subjects. A total of 31 infants with biliary atresia and 20 healthy subjects were recruited.

**Results:**

The composition of gut microbiota in BA group was significantly different with the normal control group (*P* < 0.05) and the abundance ratio of *Klebsiella/Bifidobacterium* showed great potential for identification of BA (*P* < 0.01). In addition, the differential bacterial taxa were involved in lipid and vitamins metabolism.

**Conclusion:**

Our results could provide potential non-invasive biomarker for identification of biliary atresia and contribute to the treatment in terms of ameliorating microbiota dysbiosis.

**Supplementary Information:**

The online version contains supplementary material available at 10.1186/s12887-024-04582-9.

## Introduction

Biliary atresia (BA) is a rare but life-threatening neonatal disease [1]. BA is characterized by progressive intra- and extra-hepatic bile duct inflammation and liver fibrosis, which eventually develops into liver cirrhosis and liver failure [2]. Without appropriate treatment, the median overall survival is less than two years after diagnosis [3]. At present, Kasai portoenterostomy (KPE) is recognized as an effective way to treat BA [4, 5]. However, even after successful KPE surgery, more than 75% of patients eventually have to undergo liver transplantation for end-stage liver disease [6–8]. As the most common complication after KPE, cholangitis can be as high as 40-93% and is an important factor for poor prognosis [9–11]. Considering the serious adverse consequences, early diagnosis and treatment are prominent for children with BA.

Although cholangiography is broadly applied as the gold standard of diagnosis, it is an invasive procedure. Some new potential biomarkers for diagnosis of BA have been reported. Yang et al. [12] found that serum matrix metalloproteinase-7 (MMP-7) had high sensitivity and specificity in differentiating BA from other infants with cholestasis, and might be a biomarker for the diagnosis of BA. Serum interleukin-18 (IL-18), IL-33 and γ-glutamyl transterase (GGT) were useful biomarkers for the diagnosis of BA [13]. Triangular cord sign at the porta hepatis combined with an absent or abnormal gallbladder is also an important feature of BA on the ultrasound images [14].

The etiology of BA is still unclear, which may be related to viral infection, genetic abnormalities [15], and abnormal autoimmune function [7]. As a virtual organ of human body, the gut microbiota not only plays a role in host material metabolism, energy transformation, immune response and other activities, but also closely related to the host’s health and disease status [16]. It has been reported that the dysbiosis of gut microbiota is closely related to the occurrence of autoimmune disease, such as inflammatory bowel disease (IBD) [17] and systemic lupus erythematosus (SLE) [18].

The recently discovered dysbiosis in BA infants has attracted growing interest for comprehensive analysis of gut microbiome. Wang et al. [19] reported that gut microbiota dysbiosis was related to the liver function and had a good diagnostic potential for BA. Wessel et al. [20] found the composition of gut microbiota in the BA neonates before surgery was different from that in the control group, and it was also related to the postoperative jaundice clearance. However, there are few studies on gut microbiota and diagnosis of BA.

In this study, 16S rRNA gene sequencing was carried out to identify the differences in composition and diversity of gut microbiota between infants with BA and healthy subjects, also explore the relationship between gut microbiome dysbiosis and identification of BA. Our findings suggested a strong correlation between BA and alterations of gut microbiome and *Klebsiella/Bifidobacterium* could be used to develop a non-invasive biomarker for identification of BA.

## Materials and methods

### Patient enrollment

The study population included 31 infants with BA and 20 healthy subjects, all of whom were enrolled at the Guangzhou Women and Children’s Medical Center from June 2020 to July 2021. The diagnostic criteria of biliary atresia include: (1) delayed regression and continuous aggravation of postnatal jaundice; (2) stool color gradually becomes light to acholic, and the color of urine deepens to dark; (3) abdominal distension, hepatosplenomegaly, abdominal varicose veins; (4) direct bilirubin fraction is greater than 20% of the total; (5) B-ultrasound showed that the gallbladder was strip-shaped or no cystic cavity, and the gallbladder volume did not change before and after eating. All the BA patients who were finally confirmed by operative cholangiography and the surgical age less than 120 days were included in this study. Patients with acute cholangitis, liver failure, malignancy, or other diseases that cannot tolerate surgery were excluded from this study. In addition, infants with birth weight less than 1500 g, cholestasis caused by drugs or total parenteral nutrition, bacterial or viral infection, or hemolytic diseases were also excluded from this study. All infants had no history of oral antibiotics. There was no infection or liver disease in the normal control group, and those who had taken antibiotics 2 months prior to study enrollment were excluded.

Written informed consent for BA patients was obtained on the first day after admission from all patients’ guardians before participating in the study, while the informed consent for healthy infants was obtained at the outpatient department of children health care. The protocol was approved by the Ethics Committee of Guangzhou Women and Children’s Medical Center (approval no. 401B01) and the research was conducted in compliance with the World Medical Association Declaration of Helsinki.

### Sample collection and DNA extraction

Fecal samples from BA patients were collected on the first day of admission without any treatment, while fecal samples from healthy infants were collected at the outpatient department of children health care after informed consent was obtained. All fecal samples were taken through the anus by using a sampling stick and saved in the sampling tubes with preservative solution. Fecal samples were frozen immediately at − 80 °C. The tubes and preservative solution were provided by Halgen Ltd. (Zhongshan, China). Genomic DNA was extracted from each fecal sample by QIAamp Fast DNA Stool Mini Kit (QIAGEN, Germany). The amount of DNA was quantified by Qubit® 2.0 Fluorometer (Life Technologies, USA). Integrity and size of DNA were examined by 1.0% (W/V) agarose gel electrophoresis. All DNA samples were stored at − 20 °C until further analysis.

### 16S rRNA sequencing

Sample-specific 7-bp barcodes and specific primers (F: 5’-ACTCCTACGGGAGGCAGCA-3’ and R: 5’-GGACTACHVGGGTWTCTAAT-3’) were used to amplify the V3-V4 regions of the bacterial 16 S rRNA gene from each sample. The PCR amplicons were then analyzed by gel electrophoresis and purified using Vazyme VAHTSTM DNA Clean Beads (Vazyme, Nanjing, China) and quantified using the Quant-iT PicoGreen dsDNA Assay Kit (Invitrogen, Carlsbad, CA, USA). Finally, the purified libraries were sequenced using next-generation sequencing performed on the Illumina MiSeq system using 250 bp paired-end reads.

### Bioinformatics and statistical analyses

All sequences were processed using the QIIME 2 software [https://pubmed.ncbi.nlm.nih.gov/31341288/]. After filtering the reads by quality, the operational taxonomic units (OTUs) were generated based on sequences with ≥ 97% similarity and then annotate by the GreenGenes database [https://pubmed.ncbi.nlm.nih.gov/19343057/]. Next, the α-diversity was measured by the number of observed species and the Chao index. The β-diversity was measured via unweighted unifrac and Bray-curtis distance matrix and PERMANOVA test was performed to identify significant distinction between groups. Linear discriminant analysis effect size (LEfSe) algorithm was used to discover discriminating taxa of the BA group [https://pubmed.ncbi.nlm.nih.gov/21702898/]. The PICRUSt software [https://www.ncbi.nlm.nih.gov/pmc/articles/PMC3819121/] along with the Kyoto Encyclopedia of Genes and Genomes (KEGG) database as a reference was utilized to determine the enrichment of functional pathways. Demographic and clinical data were presented as means ± standard deviation for continuous variables or percentage for categorical variables. *χ*^2^ tests were performed to compare the distribution of sex, modes of delivery and diet between the BA and normal control groups. Student’s *t*-tests were applied to compare continuous variables. A level of *P* < 0.05 was considered statistically significant. The raw sequencing data for this study can be found in the National Genomics Data Center (https://ngdc.cncb.ac.cn/ Accession number: HRA003170) database.

## Results

### Characteristics of study subjects

The study population included 31 infants with BA and 20 healthy children admitted to the Guangzhou Women and Children’s Medical Center. The demographic features of the participants were shown in Table S1. No significant difference was found between BA and control groups in terms of age, gender, gestational age, mode of delivery or feeding practice. Among the 31 infants with BA, 21 were finally treated with KPE.

### Microbiome diversity analysis

**Fig. 1:**
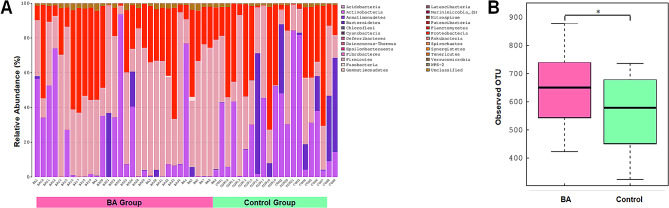
The composition of microbiota in study subjects. (**A**) Relative abundance of individual phyla. (**B**) Number of observed OTU in BA and control group. *, *p* < 0.05

To reveal the distinction of gut microbiota between the BA subjects and healthy neonates, a total of 4,783,158 sequencing tags were obtained from 51 stool samples, with an average of 93787.4 tags per sample. All tags were clustered into 4654 operational taxonomic units (OTUs). At phylum level, we observed the presence of 25 phyla across all samples (Fig. 1A). The relative abundance of *Firmicutes* and *Fusobacteria* was significantly higher in BA subjects, while the abundance of *Actinobacteria* was found to be lower than in healthy controls. The BA group is characterized by higher number of OTU as compared to the control group (Fig. 1B, *P* = 0.044).

### Distinction between BA and control groups

**Fig. 2:**
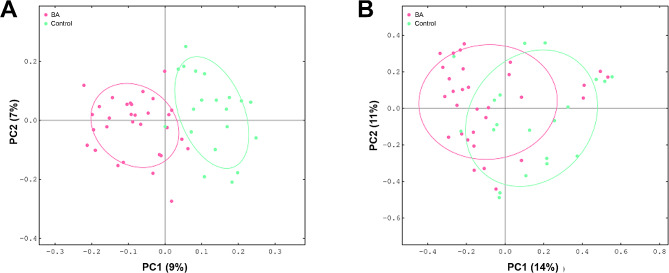
Plots of principal coordinate (PCoA) analysis. (**A**) PCoA based on unweighted Unifrac distance. (**B**) PCoA based on Bray-Curtis distance

**Fig. 3:**
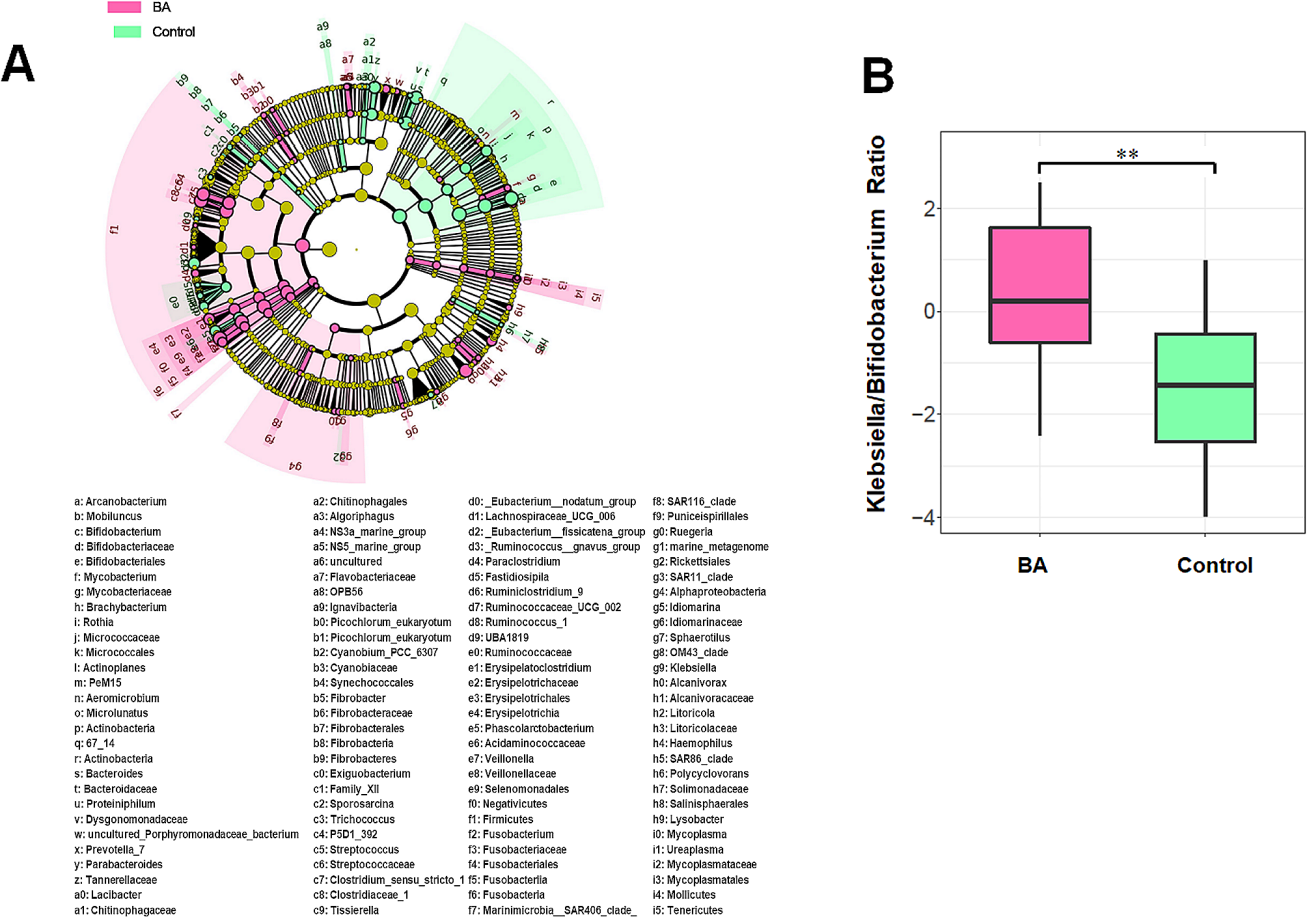
Taxonomic differences of gut microbiota between BA and control group. (**A**) Cladogram showing taxa enriched in BA group (pink) and control group (green). (**B**) Boxplot showing *Klebsiella/Bifidobacterium* ratio with significant distinction between two groups. **, *p* < 0.01

The β-diversity was assessed using unweighted Unifrac and Bray-Curtis distance and visualized Principal Coordinate Analysis (PCoA) plots. The results suggested that the gut microbiome of BA subjects clustered apart from that of healthy neonate (Fig. 2A and B, PERMANOVA *P* < 0.05). The LEfSe analysis was carried out to identify differentially abundant taxa between BA and control groups (Fig. 3A). Particularly at genus level, we observed that *Streptococcus*, *Klebsiella*, *Veillonella* were highly abundant in the BA group, while *Bifidobacterium*, *Ruminococcus*, *Rothia* were significantly less abundant. It was worth mentioning that we found the abundance ratio of *Klebsiella/Bifidobacterium* has significant difference in differentiating BA from normal control group (*P* < 0.01) (Fig. 3B).

**Fig. 4:**
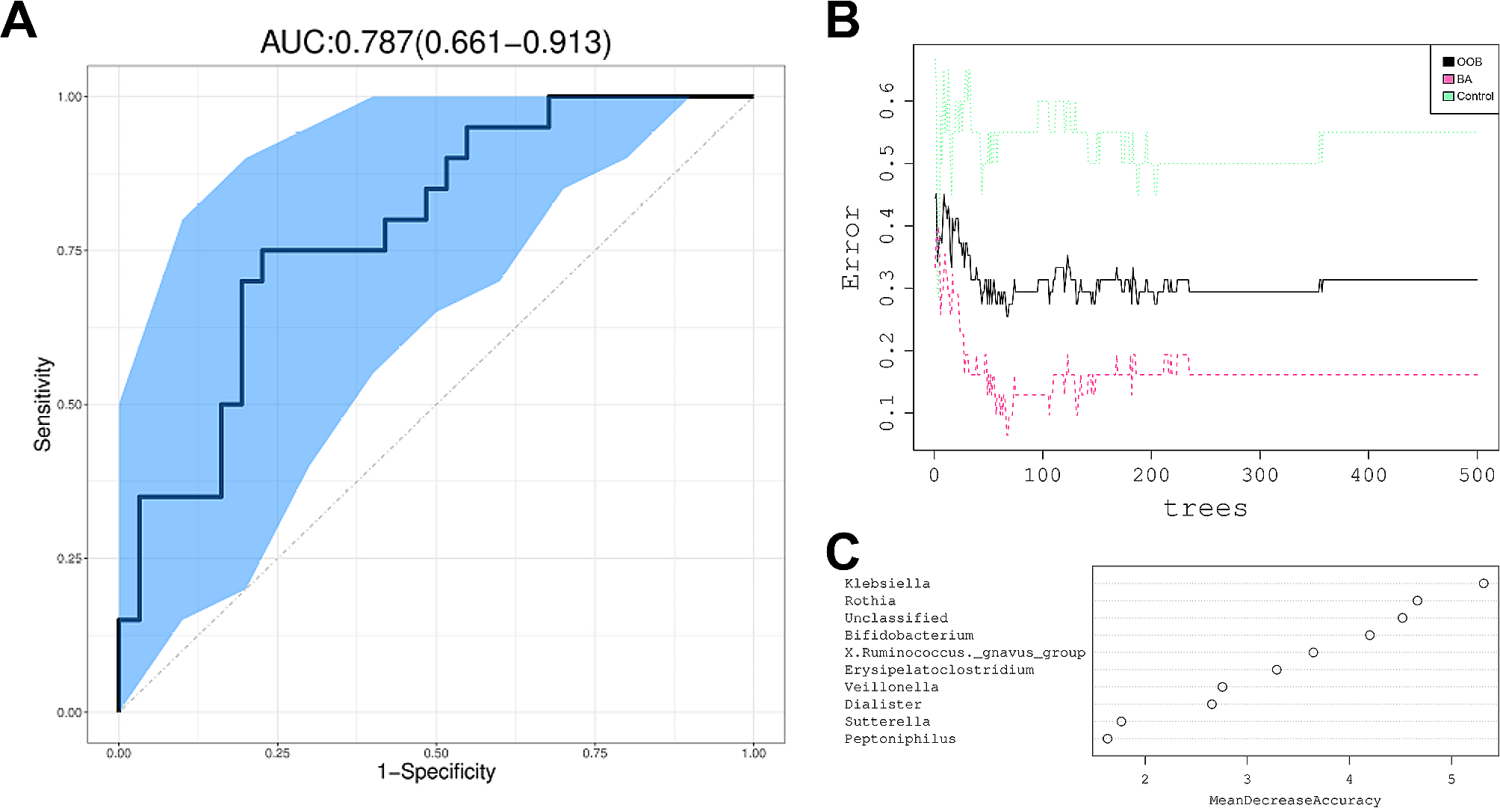
Classification of BA status by abundance of bacterial genera. (**A**) Classification performance of the random forest model was assessed by area under the ROC curve. (**B**) Error rate corresponding to tree numbers in the random forest model. OOB = out-of-bag. (**C**) The top 10 discriminant genera in the model

To test whether the microbiota characteristics can discriminate BA status, we trained a random forest model with genus abundance data. The performance of model was evaluated following the leave-one-out cross-validation (LOOCV) procedure and measured by a receiver operating characteristic (ROC) curve. Given the area under the ROC curve (AUC), we found that BA neonates were efficiently discriminated by the genus-level model with AUC = 0.787 (95% CI: 0.661–0.913, Fig. 4A-B). The major predictive power was dependent on the abundance of several genera, such as *Klebsiella*, *Rothia*, and *Bifidobacterium* (Fig. 4C).

### Functional annotation of significantly changed bacterial taxa

**Fig. 5:**
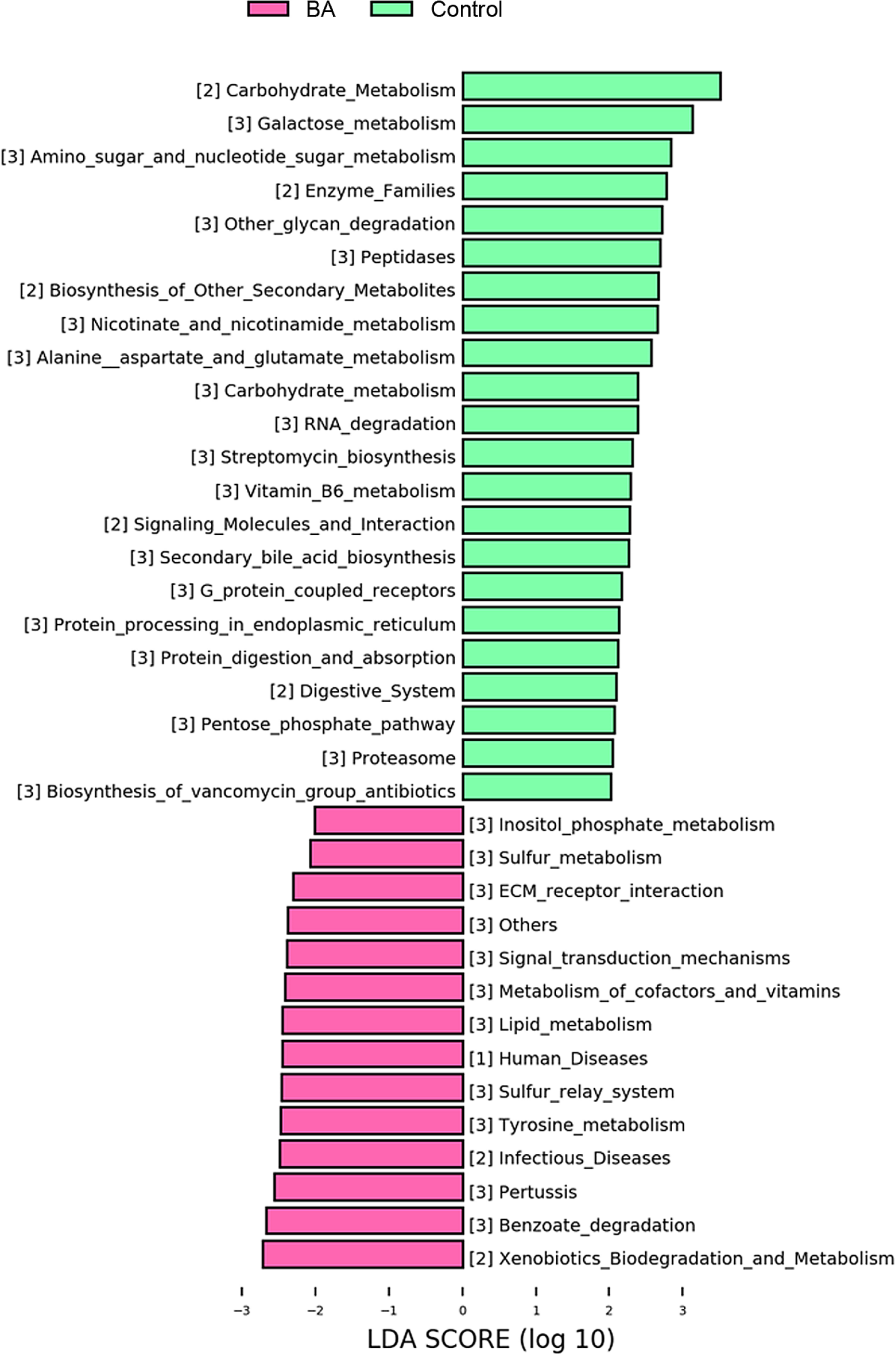
KEGG enrichment analysis of differential bacterial taxa. LDA = linear discriminant analysis

To gain a deeper understanding of the relationship between BA and gut microbiome dysbiosis, the PICRUSt software was used to pinpoint the relevant KEGG pathways (see Materials and Methods). The categories with differential enrichment between the BA and control group were involved in lipid and vitamins metabolism (Fig. 5). These functional pathways collectively indicated that gut microbiome dysbiosis was involved in the lipid and vitamins metabolism in affected infants.

## Discussion

Biliary atresia is an obstructive jaundice disease that seriously endangers the life of infants. Early diagnosis is crucial for the treatment of biliary atresia. Studies have shown that stool color card and MMP-7 can be used as a tool and biomarker for early screening and diagnosis of BA [12, 21]. Our study found the abundance ratio of *Klebsiella/Bifidobacterium* in BA was significantly different from normal control group, which can be used as a tool for identification of BA.

Gut microbiota is closely related to feeding practice and delivery modes. Forbes et al. [22] found breastfeeding can play a protective role through gut microbiota to avoid overweight while formula feeding was associated with overweight by changing the composition of gut microbiota. Caesarean section (C-section) also has a significant impact on the gut microbiota of infants, which is associated with a lower abundance of *Bifidobacteria* and a high abundance of opportunistic pathogens [23]. In this study, there was no significant difference between BA group and normal control group in the modes of delivery and feeding, thus avoiding the impact of both on the composition of gut microbiota.

Previous research has provided preliminary evidence that gut microbiota dysbiosis may play a non-negligible role in the pathogenesis of BA [20]. In the present study, we further corroborated that infants with BA exhibited unique characteristics of gut microbiota. The current results provided important new evidence that gut microbiota in BA subjects was significantly different from that of healthy individuals, which was characterized by the differential abundance of multiple bacterial taxa. Moreover, our results suggested that the abundance ratio of *Klebsiella/Bifidobacterium* can used to build up discriminative model and distinguish BA cases from healthy subjects. These findings pointed to the possibility to develop accurate biomarkers to help prevention and clinical management of BA.

Comparing our findings and previously published results, we noticed that the composition of gut microbiota in BA patients was indeed significantly different from that in normal children. Compared with the normal control group, the gut microbiota in BA group showed lower diversity and significant structural segregation [19, 24]. At the phylum level, the numbers of *Firmicutes* and *Proteobacteria* increased, while the number of *Bacteroidetes* decreased in BA. *Streptococcus* and *Klebsiella* proliferated in BA, while the numbers of *Bifidobacteria* and *Butyric*-producing bacteria decreased [19, 20]. The abundance ratio of *Streptococcus/Bacteroides* showed great potential in distinguishing BA group from normal control group [19]. It was worth mentioning that our study also found that the abundance of *Firmicutes* and *Fusobacteria* was significantly higher in BA subjects, while the abundance of *Actinobacteria* was found to be lower than in healthy controls. In addition, the abundance difference of related bacteria, such as *Klebsiella*, *Rothia* and *Bifidobacterium*, was the key factor to distinguish BA from normal control group. More importantly founding was the abundance ratio of *Klebsiella/Bifidobacterium* showed great potential for identification of BA. Our study also found that compared with the normal control group, *Veillonella* increased significantly in BA patients, while the abundance of *Ruminococcus* and *Rothia* decreased significantly. These results have not been reported in the literature and need to be confirmed by large sample size studies in future. Sun et al. [25] also reported that the composition of gut microbiota is different between biliary atresia and non-biliary atresia cholestasis. Whether the abundance ratio of *Klebsiella/Bifidobacterium* can serve as a tool to distinguish BA and cholestasis will be studied in future work.

Recent studies have shown that there is an interaction between gut microbiota and bile acid homeostasis [24]. Bile acid has a protective effect on intestinal mucosal barrier. However, the drainage of bile acid is limited in children with BA, which may cause gut microbiota dysbiosis. Conversely, the imbalance of gut microbiota will also affect bile drainage, further affecting the postoperative outcome of children with BA. Tessier et al. [24] found that high abundance of *Bifidobacterium* was associated with very good bile flow (VGBF) and reduced cholestasis 1 month after KPE. Chen et al. [26] confirmed that there was a significant amount of *Bifidobacteria* in feces of patients with normal liver function and gradual removal of jaundice 6 weeks after KPE. Wessel et al. [20] showed that compared with the clearance of jaundice (COJ)(-) group, the abundance of *Acinetobacter* and *Clostridium* was higher in COJ(+) group, while the abundance of *Enterobacter* was lower, suggesting that the composition of gut microbiota before KPE in BA patients was related to postoperative jaundice clearance. The results of our study showed that compared with the normal control group, the differences in the gut microbiota of BA patients were mainly involved in lipid metabolism and vitamin metabolism. As gut microbiota can deconjugate and transform intestinal bile acids, and then affect the signal transduction and production of bile acids in liver, so we speculated that gut microbiome dysbiosis might influence the outcome of BA patients by regulating bile drainage.

However, there were several limitations to this study. First of all, the small sample size restricted the power of statistical analysis. Second, this study was conducted in Guangzhou, China with a skew in racial and demographic characteristics. The results may not necessarily represent individuals from other areas. Third, this study was only a correlation study on the relationship between gut microbiota and the occurrence of BA. Further studies are required to recruit additional patient from multiple sites.

## Conclusions

In summary, this study makes the case that alterations of gut microbiota are associated with the incidence of biliary atresia. *Klebsiella*/*Bifidobacterium* could be used as a biomarker for identification of biliary atresia. Gut microbiome dysbiosis was involved in the lipid and vitamins metabolism in BA. The specific interaction of the gut microbiome dysbiosis and biliary atresia should be validated in further prospective studies on large cohorts.

### Electronic supplementary material

Below is the link to the electronic supplementary material.

Supplementary Material 1

Supplementary Material 2

## Data Availability

The raw sequencing data for this study can be found in the National Genomics Data Center (https://ngdc.cncb.ac.cn/ Accession number: HRA003170) database. The data and material used or analysed during the current study are available from the corresponding author on reasonable request.

## Abbreviations

BA: Biliary atresia
KPE: Kasai portoenterostomy
MMP-7: Matrix metalloproteinase-7
IL-18: Interleukin-18
GGT: γ-glutamyl transterase
IBD: Inflammatory bowel disease
SLE: Systemic lupus erythematosus
OTUs: Operational taxonomic units
LEfSe: Linear discriminant analysis effect size
KEGG: Kyoto Encyclopedia of Genes and Genomes
PCoA: Principal Coordinate Analysis
LOOCV: Leave-one-out cross-validation
ROC: Receiver operating characteristic
AUC: Area under curve
VGBF: Very good bile flow
COJ: Clearance of jaundice
