# Supplementary material for: Alterations of gut microbiota in infants with biliary atresia identified by 16S rRNA-sequencing
Source: BMC Pediatr. 2024 Feb 14;24:117. doi: 10.1186/s12887-024-04582-9 (PMC10865691; doi:10.1186/s12887-024-04582-9)
Supplement: Supplementary file 1 — Supplementary Material 1 [file 12887_2024_4582_MOESM1_ESM.pdf]

Table 1. Basic characteristics of study participants

	**BA group (n=31)**	**Control group (n=20)**	***P*-value**
**Age** [day, median(range)]	70 (39-109)	74 (32-119)	0.470
**Gender**			0.813
Male (n,%)	16 (51.6%)	11 (55%)	
Female (n,%)	15 (48.4%)	9 (45%)	
**Gestational age** [week, median(range)]	38.6 (35-40)	39.2 (37-41)	0.087
**Mode of delivery**			0.767
Spontaneous delivery (n,%)	20 (64.5%)	14 (70%)	
Cesarean section (n,%)	11 (35.5%)	6 (30%)	
**Feeding practice**			0.636
Breastfeeding (n,%)	27 (87.1%)	19 (95%)	
Non-breastfeeding (n,%)	4 (12.9%)	1 (5%)	
