# Supplementary material for: Alterations of gut microbiota in infants with biliary atresia identified by 16S rRNA-sequencing
Source: BMC Pediatr. 2024 Feb 14;24:117. doi: 10.1186/s12887-024-04582-9 (PMC10865691; doi:10.1186/s12887-024-04582-9)
Supplement: Supplementary file 2 — Supplementary Material 2 [file 12887_2024_4582_MOESM2_ESM.pdf]

Supplementary Data1. Comparison of different microbiota between BA and Control group

**Taxonomy**	**BA**	**CON**	***p*-value**
Bacteria-Firmicutes-Negativicutes-Selenomonadales-Veillonellaceae-Veillonella	18.24499	7.46915	0.0206
Bacteria-Proteobacteria-Gammaproteobacteria-Xanthomonadales-Xanthomonadaceae-Lysobacter	0.001398	0.000684	0.01444
Bacteria-Bacteroidetes-Bacteroidia-Bacteroidales-Prevotellaceae-Prevotella_7	0.02076	0.012321	0.04386
Bacteria-Actinobacteria-Actinobacteria-Bifidobacteriales-Bifidobacteriaceae-Bifidobacterium	17.92498	28.00987	0.03547
Bacteria-Proteobacteria-Alphaproteobacteria-Rhodobacterales-Rhodobacteraceae-Ruegeria	0.002159	0.000196	0.00019
Bacteria-Actinobacteria-Actinobacteria-Micrococcales-Micrococcaceae-Rothia	0.081694	0.738039	0.01898
Bacteria-Bacteroidetes-Bacteroidia-Flavobacteriales-Flavobacteriaceae-uncultured	0.002146	0.000251	0.00011
Bacteria-Firmicutes-Negativicutes-Selenomonadales-Acidaminococcaceae-Phascolarctobacterium	0.000843	0.552716	0.01817
Bacteria-Firmicutes-Bacilli-Lactobacillales-Streptococcaceae-Streptococcus	5.745462	2.347618	0.03382
Bacteria-Bacteroidetes-Bacteroidia-Chitinophagales-Chitinophagaceae-Lacibacter	0	0.000666	0.00001
Bacteria-Firmicutes-Clostridia-Clostridiales-Ruminococcaceae-Fastidiosipila	2.95E-05	0.000416	0.0435
Bacteria-Actinobacteria-Actinobacteria-Micrococcales-Dermabacteraceae-Brachybacterium	0	0.00038	0.0103
Bacteria-Firmicutes-Clostridia-Clostridiales-Peptostreptococcaceae-Paraclostridium	0.014017	0.000443	0.00828
Bacteria-Bacteroidetes-Bacteroidia-Bacteroidales-Dysgonomonadaceae-Proteiniphilum	0	0.000273	0.0103
Bacteria-Bacteroidetes-Bacteroidia-Cytophagales-Cyclobacteriaceae-Algoriphagus	0.000391	0.001464	0.00265
Bacteria-Proteobacteria-Gammaproteobacteria-Pasteurellales-Pasteurellaceae-Haemophilus	0.229717	0.029224	0.04331
Bacteria-Tenericutes-Mollicutes-Mycoplasmatales-Mycoplasmataceae-Mycoplasma	0.015583	0.001434	0.00029
Bacteria-Fibrobacteres-Fibrobacteria-Fibrobacterales-Fibrobacteraceae-Fibrobacter	0	0.000178	0.02783
Bacteria-Bacteroidetes-Bacteroidia-Bacteroidales-Tannerellaceae-Parabacteroides	0.019712	2.441066	0.04059
Bacteria-Proteobacteria-Gammaproteobacteria-Betaproteobacteriales-Burkholderiaceae-Sphaerotilus	0.000151	0.001045	0.00034
Bacteria-Firmicutes-Clostridia-Clostridiales-Ruminococcaceae-Ruminiclostridium_9	0.000549	0.001417	0.01251
Bacteria-Bacteroidetes-Bacteroidia-Flavobacteriales-Flavobacteriaceae-NS5_marine_group	0.002813	0.000357	0.00025
Bacteria-Actinobacteria-Actinobacteria-Propionibacteriales-Nocardioidaceae-Aeromicrobium	0.000728	0.000181	0.02751
Bacteria-Firmicutes-Clostridia-Clostridiales-Clostridiaceae_1-Clostridium_sensu_stricto_1	16.52042	11.02265	0.03382

Bacteria-Bacteroidetes-Bacteroidia-Flavobacteriales-Flavobacteriaceae-NS3a_marine_group	0.000485	6.67E-05	0.02695
Bacteria-Actinobacteria-Actinobacteria-Propionibacteriales-Propionibacteriaceae-Microlunatus	0.000416	0.000595	0.0211
Bacteria-Actinobacteria-Actinobacteria-Micromonosporales-Micromonosporaceae-Actinoplanes	0.000533	6.67E-05	0.01822
Bacteria-Proteobacteria-Gammaproteobacteria-Salinisphaerales-Solimonadaceae-Polycyclovorans	0	0.000205	0.02783
Bacteria-Bacteroidetes-Bacteroidia-Bacteroidales-Muribaculaceae-uncultured_Porphyromonadaceae_bacterium	0.00097	0.000459	0.04422
Bacteria-Proteobacteria-Gammaproteobacteria-Oceanospirillales-Alcanivoracaceae-Alcanivorax	0.000696	0	0.00184
Bacteria-Cyanobacteria-Oxyphotobacteria-Chloroplast-Picochlorum_eukaryotum-Picochlorum_eukaryotum	0.000404	0	0.00538
Bacteria-Firmicutes-Bacilli-Bacillales-Family_XII-Exiguobacterium	0.000441	0.000736	0.01526
Bacteria-Firmicutes-Erysipelotrichia-Erysipelotrichales-Erysipelotrichaceae-Erysipelatoclostridium	0.955439	0.125967	0.00428
Bacteria-Bacteroidetes-Bacteroidia-Bacteroidales-Bacteroidaceae-Bacteroides	1.99755	9.507169	0.0092
Bacteria-Tenericutes-Mollicutes-Mycoplasmatales-Mycoplasmataceae-Ureaplasma	0.00027	0	0.03857
Bacteria-Proteobacteria-Gammaproteobacteria-Enterobacteriales-Enterobacteriaceae-Klebsiella	11.7478	6.214635	0.00336
Bacteria-Actinobacteria-Actinobacteria-Actinomycetales-Actinomycetaceae-Mobiluncus	0	0.00041	0.02783
Bacteria-Actinobacteria-Actinobacteria-Actinomycetales-Actinomycetaceae-Arcanobacterium	0.000759	0.000137	0.04218
Bacteria-Firmicutes-Clostridia-Clostridiales-Family_XI-Tissierella	0	0.000511	0.00376
Bacteria-Firmicutes-Bacilli-Bacillales-Planococcaceae-Sporosarcina	0.000207	0.001069	0.04129
Bacteria-Firmicutes-Bacilli-Lactobacillales-Carnobacteriaceae-Trichococcus	3.32E-05	0.000462	0.04753
Bacteria-Proteobacteria-Gammaproteobacteria-Alteromonadales-Idiomarinaceae-Idiomarina	0.001073	4.57E-05	0.01639
Bacteria-Proteobacteria-Gammaproteobacteria-Oceanospirillales-Litoricolaceae-Litoricola	0.001246	4.30E-05	0.00086
Bacteria-Firmicutes-Clostridia-Clostridiales-Ruminococcaceae-UBA1819	0.000198	0.049923	0.00818
Bacteria-Firmicutes-Clostridia-Clostridiales-Ruminococcaceae-Ruminococcus_1	0.000315	0.00174	0.00089
Bacteria-Fusobacteria-Fusobacteriia-Fusobacteriales-Fusobacteriaceae-Fusobacterium	0.106368	0.018029	0.03136
Bacteria-Actinobacteria-Actinobacteria-Corynebacteriales-Mycobacteriaceae-Mycobacterium	0.002693	0.00053	0.00816
Bacteria-Proteobacteria-Gammaproteobacteria-Betaproteobacteriales-Methylophilaceae-OM43_clade	0.001092	0.000461	0.01445
